# Association of higher triglyceride–glucose index and triglyceride-to-high-density lipoprotein cholesterol ratio with early neurological deterioration after thrombolysis in acute ischemic stroke patients

**DOI:** 10.3389/fneur.2024.1421655

**Published:** 2024-08-21

**Authors:** Mingzhu Deng, Kangping Song, Wei Xu, Guohua He, Jue Hu, Hui Xiao, Nina Zhou, Sufen Chen, Guilan Xu, Yangping Tong, Dan Zhang, Zhen Wang, Fangyi Li

**Affiliations:** ^1^Department of Neurology, Brain Hospital of Hunan Province, The Second People’s Hospital of Hunan Province, Changsha, China; ^2^Department of Neurology, The Affiliated Changsha Central Hospital, Hengyang Medical School, University of South China, Changsha, China

**Keywords:** acute ischemic stroke, intravenous thrombolysis, triglyceride–glucose index, triglyceride-to-high-density lipoprotein cholesterol ratio, early neurological deterioration

## Abstract

**Background:**

Insulin resistance (IR) can predict the prognosis of patients suffering from cerebrovascular disorders. The triglyceride–glucose (TyG) index and triglyceride-to-high-density lipoprotein cholesterol (TG/HDL-C) ratio have been confirmed to be easy and reliable indicators of IR. However, the relationships between the TyG index or TG/HDL-C ratio and early neurological deterioration (END) after thrombolysis in patients with acute ischemic stroke (AIS) are uncertain.

**Methods:**

A retrospective analysis of 1,187 patients diagnosed with AIS who underwent intravenous thrombolysis between January 2018 and February 2024 was performed. Post-thrombolysis END was defined as an increase in the National Institutes of Health Stroke Scale (NIHSS) score of ≥4 within 24 h after thrombolysis. Logistic regression analysis was performed to explore the relationships of the TyG index and TG/HDL-C ratio with post-thrombolysis END. Receiver operating characteristic (ROC) analysis was used to assess the ability of the TyG index and TG/HDL-C ratio to discriminate post-thrombolysis END.

**Results:**

Among the 1,187 recruited patients, 179 (15.08%) were diagnosed with post-thrombolysis END, and 1,008 (84.92%) were diagnosed with non-END. A binary logistic regression model indicated that the TyG index (odds ratio [OR], 2.015; 95% confidence interval [CI] 1.964–2.414, *p* = 0.015) and TG/HDL-C ratio (OR, 1.542; 95% CI, 1.160–2.049, *p* = 0.004) were independent factors for post-thrombolysis END. The area under the curve (AUC) values for the TyG index, TG/HDL-C ratio, and TyG index combined with the TG/HDL-C ratio for post-thrombolysis END were 0.704, 0.674, and 0.755, respectively.

**Conclusion:**

This study indicates that the TyG index and TG/HDL-C ratio can be used as prognostic factors to predict post-thrombolysis END.

## Introduction

Acute ischemic stroke (AIS), which is caused by sudden arterial blockage and results in neuronal damage, is the most common type of stroke (1, 2). The preferred treatment for AIS is intravenous recombinant tissue plasminogen activator in the early phase (≤4.5 h) (3, 4). Nevertheless, a minority of patients continue to experience early neurological deterioration (END) in which neurological impairments and symptoms intensify within 24 h after thrombolysis (5). END is associated with an increased risk of mortality and morbidity, and previous studies have shown that END is relevant to unfavorable long-term outcomes in AIS patients (6, 7). Therefore, it is important to investigate the risk factors and measurable indicators of post-thrombolysis END in AIS patients.

Insulin resistance (IR) is considered the primary pathophysiology of metabolic syndrome (8), which is involved in the pathogenesis of cerebrovascular diseases, mainly through endogenous fibrinolytic dysfunction, thrombosis, elevated platelet activation, and inflammation (9). The hyperinsulinemic–euglycemic clamp test is the gold standard for assessing IR. However, the high cost of this measurement limits its wide-scale clinical applicability (10). In recent years, the triglyceride–glucose (TyG) index and triglyceride-to-high-density lipoprotein cholesterol (TG/HDL-C) ratio have been established as reliable, cost-effective, and easily accessible surrogate markers for IR (11–15). According to large cohort studies, the TyG index might be a useful IR biomarker for predicting the prognosis of stroke patients (16). A higher TyG index is associated with more severe END in AIS patients (11). Moreover, prior research has demonstrated that an elevated TyG index is associated with poor outcomes after thrombolysis (9, 17, 18). However, there are conflicting relationships between metabolic syndrome and outcomes after thrombolysis (19, 20). Therefore, the relationship between the TyG index and post-thrombolysis END deserves further investigation. The TG/HDL-C ratio is an easily accessible serum biomarker and may be utilized for assessing IR (13). Previous studies have shown significant associations between the TG/HDL-C ratio and incident cardiovascular disease (13, 21). Nevertheless, a recent large-scale cohort study revealed that there was no significant correlation between TG/HDL-C and worse cardiovascular disease outcomes (12). Furthermore, few studies have investigated the correlation between the TG/HDL-C ratio and post-thrombolysis END.

The early neurological outcome after thrombolysis is related to the long-term prognosis of patients (7, 22). The correlation between the TyG index or HDL-C ratio and post-thrombolysis END remains unclear. Therefore, we investigated the associations of the TyG index and TG/HDL-C ratio with the risk of post-thrombolysis END.

## Materials and methods

### Study design and participants

**Figure 1:**
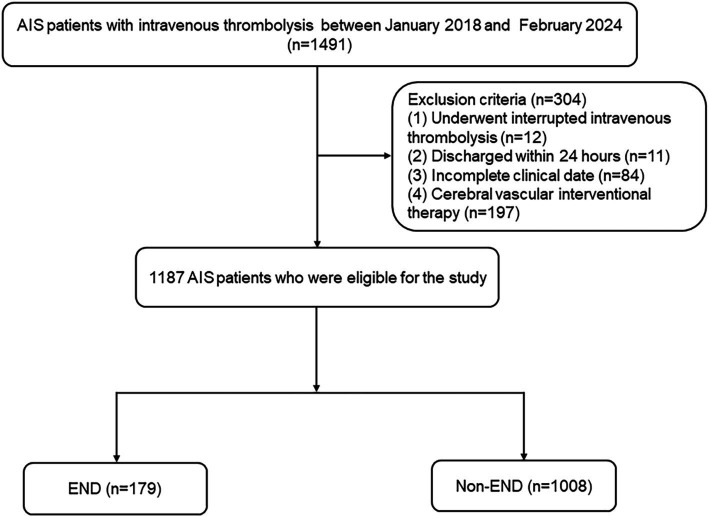
Study flow diagram. AIS, acute ischemic stroke; END, early neurological deterioration.

Patients with AIS who received intravenous thrombolysis within 4.5 h were selected from Changsha Central Hospital and Hunan Province Second People’s Hospital. AIS was diagnosed using head imaging techniques, including CT and MRI, based on the 2018 Chinese guidelines for the diagnosis and treatment of acute ischemic stroke. The diagnostic criteria were as follows: (1) acute onset; (2) focal neurological deficits (weakness or numbness of one side of the face or limb, language disorder, etc.), a few of which are global neurological deficits; (3) imaging liability lesions or symptoms/signs for more than 24 h; (4) exclusion of non-vascular causes; and (5) brain CT/MRI exclusion of cerebral hemorrhage (23). The inclusion criteria were as follows: (1) admission within 4.5 h after onset, (2) treatment with intravenous thrombolysis with r-tPA, and (3) age 18 years or older. The exclusion criteria for patients were as follows: (1) discharged within 24 h, (2) intravenous thrombolysis was interrupted due to serious side effects, (3) incomplete clinical data, and (4) cerebral vascular interventional therapy. This study was approved by the Ethics Committee of Changsha Central Hospital and Hunan Province Second People’s Hospital. Between January 2018 and February 2024, 1,187 AIS patients were recruited. Figure 1 shows a detailed flow diagram for patient enrollment.

### Data collection

Expert neurologists conducted the clinical evaluations in a blinded manner. The following information was collected from each participant: age, sex, body mass index, risk factors for stroke (hypertension, diabetes mellitus, atrial fibrillation, coronary artery disease, and current drinking and smoking), and clinical features (admission stroke onset severity, admission systolic blood pressure (SBP), diastolic blood pressure (DBP), onset to treatment time (OTT), and stroke subtypes). Computed tomography, magnetic resonance, electrocardiography, echocardiography, carotid ultrasonography, and transcranial Doppler were used to determine the stroke subtype. The stroke subtype was classified according to the Trial of Org 10172 in Acute Stroke Treatment criteria (24). Using a standardized case report form, the demographic information, baseline clinical parameters, clinical diagnoses, and treatment plans were meticulously gathered. The physicians or other healthcare professionals who had been in charge were consulted if any information was unclear.

Blood samples from all patients were collected at 6–7 a.m. on the day after the patients had fasted for at least 8 h. In total, 2 mL of EDTA-anticoagulated whole blood was used for routine blood tests (automated hematology analyzer, BZ6800, China). In total, 5 mL of coagulant-containing blood was used for standard biochemical examination (automatic analyzer, HITACHI 7600, Japan). Blood samples were evaluated for triglycerides (TG), total cholesterol (TC), low-density lipoprotein cholesterol (LDL-C), high-density lipoprotein cholesterol (HDL-C), and fasting blood glucose (FBG) levels. The TG/HDL-C ratio was calculated. Each blood test was conducted three times. The following formula was used to define the TyG index: Ln [TG (mg/dL) × FBG (mg/dL) ÷ 2] (25).

### Definition of post-thrombolysis early neurological deterioration and symptomatic intracranial hemorrhage

The two centers’ certified neurologists were blinded to our investigation and received unified training for evaluating NIHSS scores. Post-thrombolysis END was defined as an increase in the National Institutes of Health Stroke Scale (NIHSS) score by ≥4 points in the total score within 24 h after thrombolysis (26, 27). Symptomatic intracranial hemorrhage (sICH) was defined as clinical worsening of at least 4 points on the National Institutes of Health Stroke Scale (NIHSS) score within 24 h after thrombolysis, attributed to parenchymal hematoma, subarachnoid, or intraventricular hemorrhage (28).

### Statistical analysis

SPSS 25.0 (IBM SPSS Statistics software, Version 25.0) was used for data analysis. The Kolmogorov–Smirnov test was used to determine if all the data had a normal distribution. If the continuous variables were regularly distributed, they are shown as the means ± SDs; if not, they are shown as medians (quartiles). For categorical factors, the results are shown as percentages. The chi-square test or Fisher’s exact test was used for categorical variables, and Student’s *t*-test or the Mann–Whitney U test was used to evaluate differences in the baseline characteristics of continuous variables across groups. Logistic regression analysis was used to detect risk factors for post-thrombolysis END. A MedCalc 15.6.0 (MedCalc Software Acacialaan 22, B-8400 Ostend, Belgium) packet program was used to obtain a receiver operating characteristic (ROC) curve to test the overall ability of the TyG index and TG/HDL-C ratio to discriminate post-thrombolysis END. A two-tailed value of *p* < 0.05 was considered significant.

## Results

### Clinical and demographic characteristics of AIS patients with post-thrombolysis END and non-END

**Figure 2:**
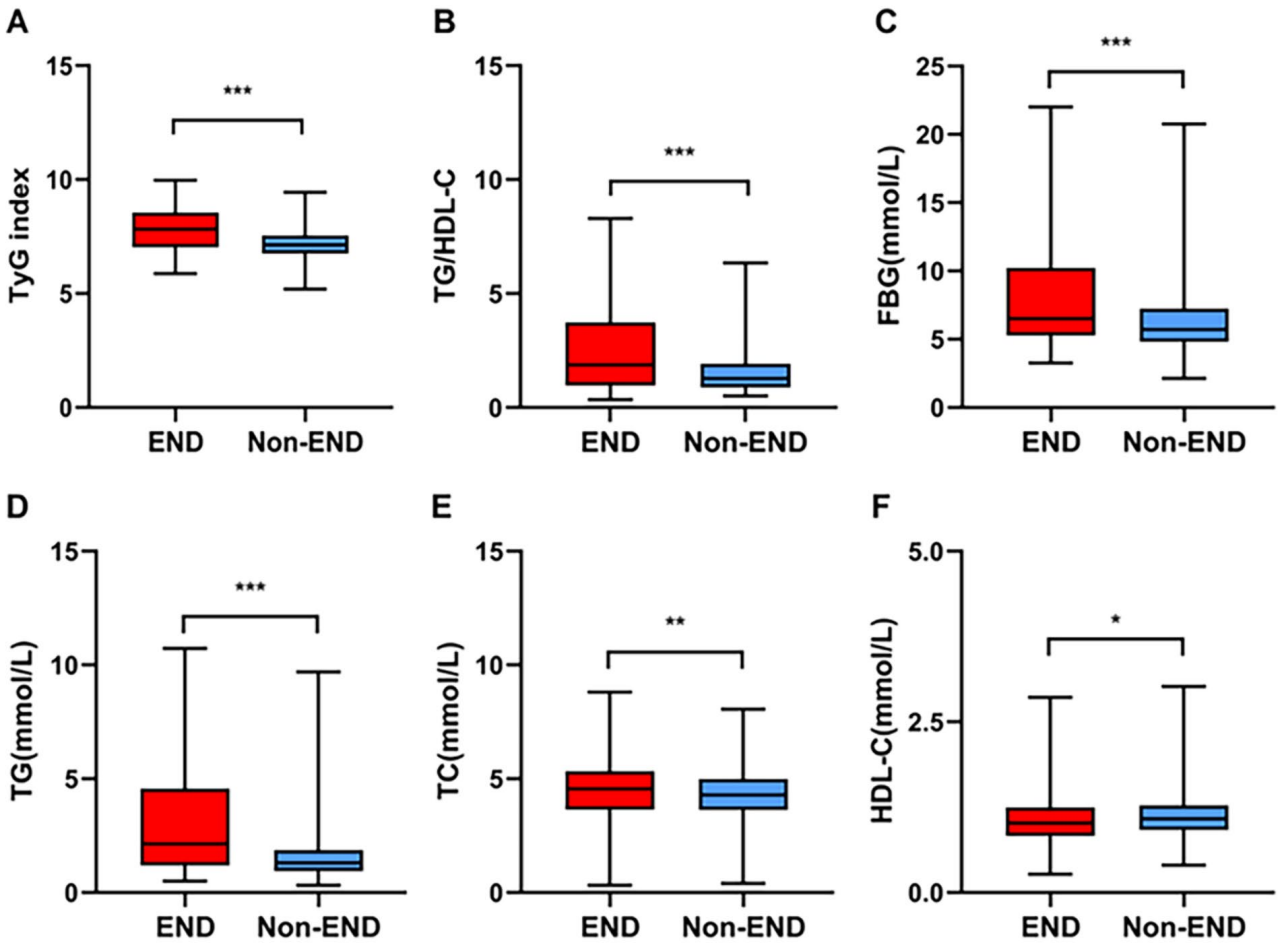
Comparisons of the TyG index **(A)**, TG/HDL-C **(B)**, FBG **(C)**, TG **(D)**, TC **(E)**, and HDL-C **(F)** between the END and non-END groups. ***p < 0.001, **p < 0.01, *p < 0.05.

Table 1 shows the clinical and demographic characteristics of the patients in detail. The baseline characteristics of the AIS patients from the two hospitals are shown in Supplementary Table S1. In our study, post-thrombolysis END was observed in 179 patients (15.08%), and post-thrombolysis non-END was observed in 1008 patients (84.92%). In the post-thrombolysis END group, the NIHSS score after rt-PA for 24 h (*p* < 0.001), DBP (*p* = 0.026), diabetes mellitus (*p* = 0.014), FBG (*p* < 0.001), TG (*P* = <0.001), TC (*p* = 0.003), TyG index (*p* < 0.001), and TG/HDL-C ratio (*p* < 0.001) were significantly greater than those in the post-thrombolysis non-END group, whereas HDL-C (*p* = 0.012) was significantly lower than those in the post-thrombolysis non-END group. In the post-thrombolysis END group, the percentage of patients with symptomatic intracranial hemorrhage (sICH) was 26.82% (48/179). In addition, stroke subtype (*p* = 0.011) was significantly different between the two groups. Figure 2 shows the TyG index, TG/HDL-C ratio, FBG, TG, TC, and HDL-C for the two groups.

**Table 1:** Characteristics of AIS patients with post-thrombolysis END and non-END patients.

Variable	END (*n* = 179)	Non-END (*n* = 1,008)	T/Z	*P*
**Demographic characteristics**				
Age, years	67.32 ± 12.18	66.39 ± 12.40	0.094	0.348
Male, *n* (%)	118 (65.92)	622 (61.71)	1.515	0.283
BMI, kg/m^2^	23.16 ± 4.75	22.99 ± 4.10	−0.434	0.665
**Clinical assessment**				
NIHSS, score at admission	6.5(3–12.25)	6 (3–12)	−0.711	0.477
NIHSS, score after rt-PA 24 h	8 (5–15)	3 (1–8)	−8.580	<0.001
sICH, *n* (%)	48 (26.82)			
SBP, mmHg	151.36 ± 24.23	147.66 ± 20.30	−1.386	0.167
DBP, mmHg	86.85 ± 13.44	83.17 ± 12.88	−2.227	0.026
OTT, minute	154 (110.5, 215)	146 (86, 213)	−1.129	0.259
**Vascular risk factors, *n* (%)**				
Hypertension	111 (62.01)	674 (66.87)	1.599	0.206
Diabetes mellitus	49 (27.37)	195 (19.35)	6.001	0.014
Atrial fibrillation	26 (14.53)	163 (16.17)	0.307	0.579
Coronary artery disease	38 (21.23)	218 (21.63)	0.014	0.905
Current smoking	77 (43.02)	406 (40.28)	0.473	0.492
Current drinking	36 (20.11)	223 (22.12)	0.360	0.548
**Medication use history, *n* (%)**				
Previous antiplatelet	20 (11.17)	145 (14.38)	1.310	0.252
Previous anticoagulation	13 (7.26)	71 (7.04)	0.011	0.916
Previous statin	11 (6.15)	76 (7.54)	0.435	0.509
Previous antihypertension	69 (38.55)	405 (40.18)	0.800	0.371
Previous hypoglycemic agents	24 (13.41)	117 (11.61)	0.471	0.493
**Stroke subtype, *n* (%)**			13.014	0.011
LAA	76 (42.46)	320 (31.75)		
SAO	68 (37.99)	476 (47.22)		
CE	22 (12.29)	168 (16.67)		
SOE	4 (2.23)	10 (0.99)		
SUE	9 (5.03)	34 (3.37)		
**Laboratory data**				
FBG (mmol/L)	6.53 (5.28–10.23)	5.71 (4.81–7.23)	−5.220	<0.001
TG (mmol/L)	1.88(1.15–3.9)	1.29 (0.94–1.82)	−7.617	<0.001
TC (mmol/L)	4.61 (3.78–5.39)	4.33 (3.7–5.01)	−2.996	0.003
HDL-C (mmol/L)	1.06 ± 0.32	1.12 ± 0.32	2.510	0.012
LDL-C (mmol/L)	2.76 ± 1.02	2.74 ± 0.88	−0.324	0.746
TyG index	7.82 (7.02–8.56)	7.13 (6.75–7.54)	−8.728	<0.001
TG/HDL-C	2.01 (0.99–4.56)	1.20 (0.80–1.86)	−7.442	<0.001

BMI, body mass index; sICH, symptomatic intracranial hemorrhage; SBP, systolic blood pressure; DBP, diastolic blood pressure; NIHSS, National Institutes of Health Stroke Scale; OTT, onset to treatment time; LAA, large-artery atherosclerosis; SAO, small-artery occlusion; CE, cardioembolism; SOE, stroke of other determined etiology; SUE, stroke of undetermined etiology; TC, total cholesterol; TG, triglycerides; FBG, fasting blood glucose; HDL-C, high-density lipoprotein cholesterol; LDL-C, low-density lipoprotein cholesterol; TyG, triglyceride–glucose.

### Logistic regression analysis for risk factors for post-thrombolysis END

**Figure 3:**
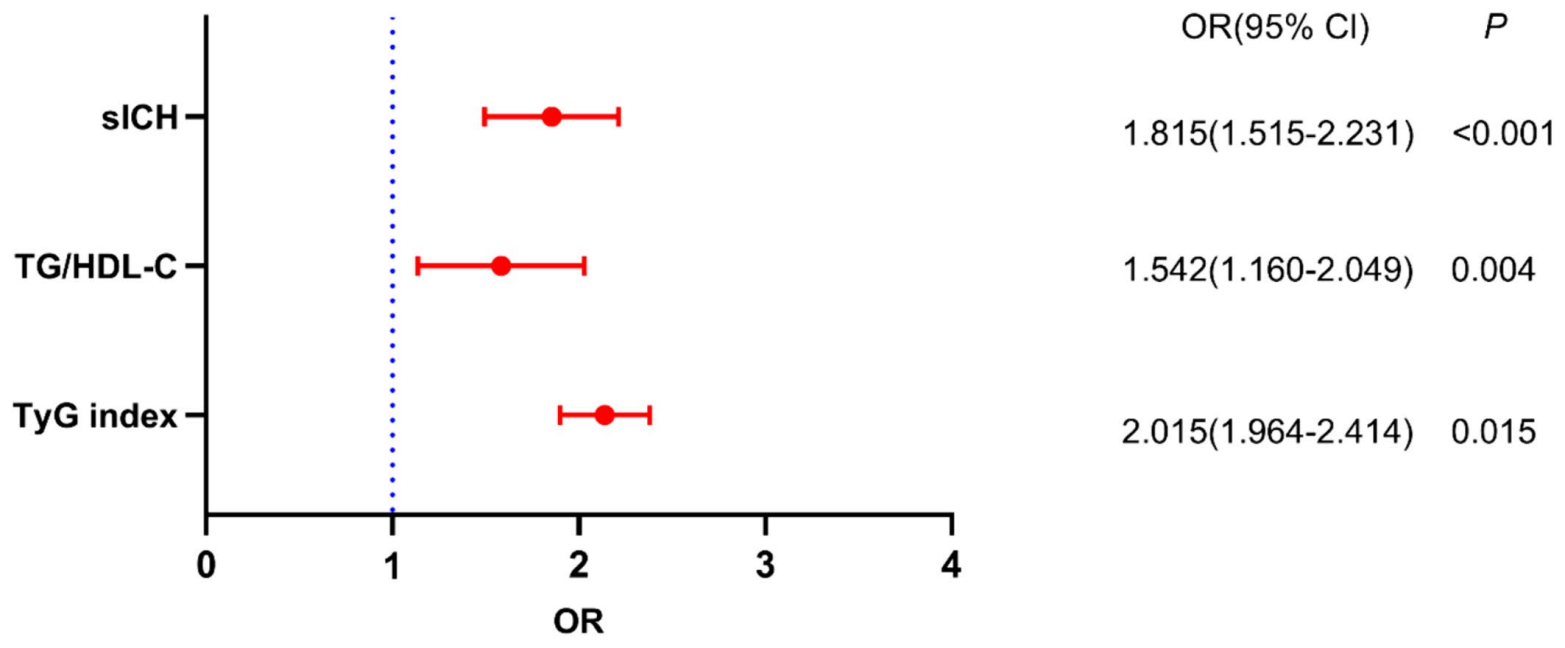
Binary logistic analysis of independent risk factors associated with post-thrombolysis END.

The results of the crude models for post-thrombolysis END are displayed in Table 2. To identify independent risk factors for post-thrombolysis END, the binary logistic regression model included variables with statistical significance mentioned in Table 1. sICH, age, and the National Institutes of Health Stroke Scale (NIHSS) score are also important factors for post-thrombolysis END and should be included in multivariate analysis. There was no collinearity between the TyG index and the TG/HDL-C ratio. However, FBG, TG, and HDL-C were not included in the model because of collinearity with the TyG index and TG/HDL-C ratio. The TyG index (OR, 2.015; 95% CI 1.964–2.414, *p* = 0.015), TG/HDL-C ratio (OR, 1.542; 95% CI 1.160–2.049, *p* = 0.004), and sICH (OR, 1.815; 95% CI 1.515–2.231, *p* < 0.001) were identified as independent factors for post-thrombolysis END after adjustment for age, initial NIHSS score, DBP, stroke subtype, and TC (Figure 3). In addition, the TyG index (median = 7.17) and TG/HDL-C ratio (median = 1.26) were used as binary categorical variables. After adjusting for all confounding factors, the T2 subgroup was still significantly associated with post-thrombolysis END, compared to the T1 subgroup (Table 3).

**Table 2:** Logistic regression analysis for risk factors for post-thrombolysis END.

Variable	OR (95% CI)	*P*	Adjusted OR (95% CI)	*P*
Age	1.294 (1.081–1.307)	0.347	1.122 (1.041–1.255)	0.435
Initial NIHSS score	1.129 (1.098–1.302)	0.658	1.005 (0.984–1.125)	0.745
sICH	2.885 (2.312–3.519)	<0.001	1.815 (1.515–2.231)	<0.001
DBP	1.022 (1.002–1.041)	0.027	1.019 (0.985–1.131)	0.095
Diabetes mellitus	1.562 (1.085–2.246)	0.016	1.087 (0.512–2.315)	0.865
LAA	Reference		Reference	
SAO	0.602 (0.421–0.859)	0.005	0.432 (0.223–0.978)	0.062
CE	0.551 (0.331–0.918)	0.022	1.032 (0.465–2.180)	0.813
SOE	1.684 (0.514–5.515)	0.389	2.296 (0.238–6.371)	0.438
SUE	1.115 (0.513–2.422)	0.784	0.452 (0.051–1.957)	0.219
FBG	1.178 (1.123–1.235)	<0.001		
TG	2.142 (1.847–2.485)	<0.001		
HDL-C	0.442 (0.241–0.810)	0.008		
TC	1.023 (0.999–1.130)	0.04	1.012 (0.982–1.113)	0.996
TyG index	3.879 (3.029–4.968)	<0.001	2.015 (1.964–2.049)	0.015
TG/HDL-C	1.847 (1.637–2.084)	<0.001	1.542 (1.160–2.049)	0.004

**Table 3:** Association of the TyG index and TG/HDL-C ratio with post-thrombolysis END.

Variable	OR (95% CI)	*P*	Adjusted OR (95% CI)^a^	*P*
**TyG index binary classification**				
T1	Reference		Reference	
T2	2.650 (1.882–3.731)	<0.001	2.32 1(2.013–2.954)	0.004
**TG/HDL-C binary classification**				
T1	Reference		Reference	
T2	2.570 (1.828–3.614)	<0.001	2.085 (1.514–2.873)	0.02

TyG index: T1 < 7.17; T2 ≥ 7.17; TG/HDL-C: T1 < 1.26; T2 ≥ 1.26.

a Model: adjusted for DBP, stroke subtype, and TC.

### ROC curve analysis to determine the overall ability to discriminate post-thrombolysis END

**Figure 4:**
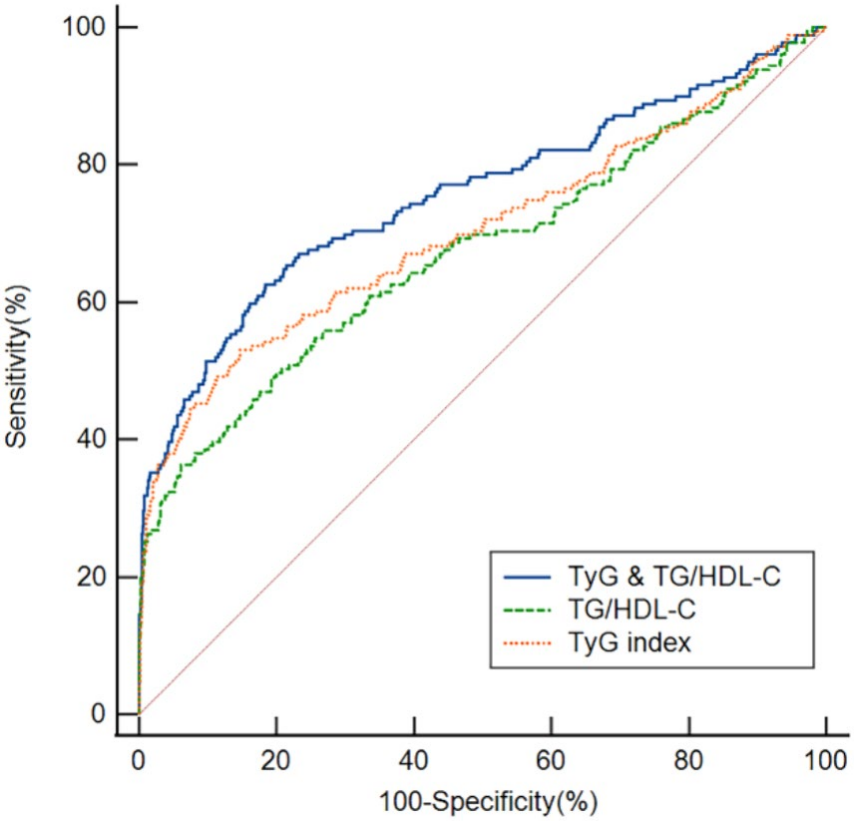
ROC analysis revealed that the TyG index, TG/HDL-C ratio, and TyG index and the TG/HDL-C ratio exhibited respectable post-thrombolysis END discriminating power, with AUC values of 0.704, 0.674, and 0.755, respectively.

We used ROC curves to assess the overall ability of the TyG index and TG/HDL-C ratio to discriminate post-thrombolysis END (Figure 4). The TyG index’s area under the curve (AUC) for determining post-thrombolysis END was 0.704 (95% CI, 0.678–0.730; *p* < 0.001), and the cut-off value was 7.78, with a sensitivity of 53.1% and a specificity of 85.3%. For the TG/HDL-C ratio, the AUC was 0.674 (95% CI, 0.647–0.701; *p* < 0.001), and the cut-off value was 2.94, with a sensitivity of 36.3% and a specificity of 93.9%. In addition, we conducted an ROC curve analysis to assess the discriminatory power of the TyG index and TG/HDL-C ratio combination in distinguishing between the END group and the non-END group. The AUC for the combination of the TyG index and TG/HDL-C ratio was 0.755 (95% CI: 0.730–0.780, *p* < 0.001), and the cut-off value was 0.18, with a sensitivity of 62.6% and a specificity of 81.6%.

## Discussion

In our research, 179 patients (15.08%) experienced post-thrombolysis END, and the proportion was consistent with the results of previous studies (5, 9, 29). The results we obtained offer a number of fresh insights. First, we found that the TyG index and TG/HDL-C ratio in AIS patients with END were greater than those in AIS patients with non-END. Second, the binary logistic regression model indicated that the TyG index and TG/HDL-C ratio were independent factors for post-thrombolysis END. Finally, we employed ROC curves to test the overall ability of the TyG index and TG/HDL-C ratio to discriminate post-thrombolysis END. Together, these findings provide evidence that a higher TyG index and TG/HDL-C ratio are associated with post-thrombolysis END.

An increasing amount of research has demonstrated a relationship between the TyG index and the prognosis of AIS patients. The TyG index is related to arterial stiffness (30) and poor outcomes in cardiovascular and cerebrovascular diseases (31, 32). According to multicenter observational research, a higher TyG index was associated with 90-day unfavorable functional outcomes in AIS patients after thrombolysis (18). Additionally, the TyG index was related to a greater risk of in-hospital mortality in patients with severe stroke (33) and early stroke recurrence (34). In our study, the TyG index was significantly greater in the post-thrombolysis END group than in the non-END group. A binary logistic regression model indicated that the TyG index was an independent factor for post-thrombolysis END. In addition, when the TyG index was used as a categorical variable, after adjusting for confounding factors, our data revealed that a higher TyG index was associated with a greater probability of developing post-thrombolysis END, which is consistent with the findings of previous studies (9). These findings may indicate that the TyG index is a biomarker for END.

The associations between post-thrombolysis END and the TyG index can be explained by several mechanisms. First, IR may cause excessive platelet activation, exacerbate endothelial dysfunction, and cause biochemical imbalances (35, 36). Second, IR induces a variety of metabolic disorders, which promote atherosclerotic plaque rupture, leading to thrombosis (37). Third, IR can exacerbate oxidative stress, which can result in the accumulation of reactive oxygen species and mitochondrial dysfunction. Finally, IR can increase matrix metalloproteinase-9 activity, which exacerbates ischemia, reperfusion damage, and the inflammatory response (38, 39).

Based on a prior study, in the hypertensive population, an increased TG/HDL-C ratio was predictive of increased risk and advanced development of arterial stiffness (40). According to a longitudinal study, the TG/HDL-C ratio may be a significant and distinct biomarker for predicting cardiovascular disease outcomes and progression (41). Furthermore, data from the UK Biobank cohort revealed that an elevated TG/HDL-C ratio was associated with a greater risk of cardiovascular disease (13). However, the correlation between the TG/HDL-C ratio and post-thrombolysis END in patients with AIS remains unclear. In this study, in the post-thrombolysis END group, the TG/HDL-C ratio was significantly greater than that in the non-END group. Furthermore, the TG/HDL-C ratio was identified as an independent factor for post-thrombolysis END, after adjustment for potential confounders. Employing the TG/HDL-C ratio as a categorical variable, our results demonstrated that a higher TG/HDL-C ratio was related to an increased risk of post-thrombolysis END development. Our findings expand the understanding of the function of the TG/HDL-C ratio in cerebrovascular disease and provide fresh perspectives on therapeutic approaches.

In our study, the proportion of sICH in the END group was 26.82% (48/179), which is similar to the study by Yu et al. (27), and the overall percentage of patients with sICH was 4.04% (48/1,187) for all AIS patients after thrombolysis, which is consistent with earlier studies (1, 28). In this research, sICH was identified as an independent risk factor for post-thrombolysis END after adjustment for all potential confounders, which is similar to the earlier study (42). Age and the NIHSS score were also important factors for END (43). However, there was no significant correlation between age, the NIHSS score, and post-thrombolysis END in our research. We believe that the variances in the ethnicity of the research populations, the sample sizes, the medication status, and the severity of the condition may be the causes of the discrepancies between various studies.

We employed ROC curves to test the overall ability of the TyG index and TG/HDL-C ratio to discriminate post-thrombolysis END. Our research revealed that the TyG index and TG/HDL-C ratio had the ability to distinguish patients in the END group from those in the non-END group. The TyG index is more discriminative than the TG/HDL-C ratio, indicating that the TyG index might be a valuable instrument for predicting post-thrombolysis END. Moreover, our study revealed that the combination of the TyG index and TG/HDL-C ratio exhibited superior discriminatory power for post-thrombolysis END, with an AUC of 0.755. This value surpassed that of the individual markers, suggesting that the combination of these two indicators may be more beneficial in predicting post-thrombolysis END.

The limitations of this study are as follows: (1) This was a cross-sectional study with only Chinese patients receiving intravenous thrombolysis; thus, potential inherent biases exist. As a result, our findings need to be verified in non-Chinese populations, and future research may require larger-scale longitudinal cohort studies. (2) Owing to realistic limitations resulting from our clinical context, our research team did not employ a direct IR assessment tool. (3) Given that IR detection is reliant on time during the onset of AIS, early measurement may have overestimated the prevalence of IR. Future research needs to consider the effects of peripheral blood markers.

## Conclusion

In conclusion, our study suggests that the TyG index and TG/HDL-C ratio can be used as prognostic factors to predict post-thrombolysis END. Additionally, the combination of the TyG index and TG/HDL-C ratio may provide greater predictive value. However, more research is needed to confirm these findings and clarify the pathophysiology of post-thrombolysis END.

## Data Availability

The raw data supporting the conclusions of this article will be made available by the authors, without undue reservation.
