# Supplementary material for: Association of higher triglyceride–glucose index and triglyceride-to-high-density lipoprotein cholesterol ratio with early neurological deterioration after thrombolysis in acute ischemic stroke patients
Source: Front Neurol. 2024 Aug 21;15:1421655. doi: 10.3389/fneur.2024.1421655 (PMC11371550; doi:10.3389/fneur.2024.1421655)
Supplement: Supplementary file 1 [file Table_1.DOCX]

**Supplementary Table 1 Baseline characteristics of AIS patients from two hospitals**

| **Variable** | **A(n=674)** | **B(n=513)** | **T/Z** | ***P*** |
| --- | --- | --- | --- | --- |
| **Demographic characteristics** |  |  |  |  |
| Age, years | 66.75±12.75 | 67.63±11.59 | -1.235 | 0.217 |
| Male, n (%) | 411(60.98) | 329(64.13) | 1.234 | 0.267 |
| BMI, kg/m^2^ | 21.94±6.63 | 21.47±6.38 | 1.066 | 0.286 |
| **Clinical assessment**  NIHSS, score at admission | 7(4-13) | 6(3-12) | -1.936 | 0.063 |
| NIHSS, score after rt-PA 24h | 5(2-11) | 4(1-10) | -2.384 | 0.195 |
| sICH, n (%) | 29(4.01) | 19(4.10) | 0.269 | 0.604 |
| SBP, mmHg | 149.95±22.32 | 148.34±21.2 | 1.743 | 0.182 |
| DBP, mmHg | 84.46±12.84 | 83.94±12.93 | 1.284 | 0.482 |
| OTT, minute | 153(105.5-216) | 149.5(92-212.75) | -2.291 | 0.003 |
| **Vascular risk factors, n (%)** |  |  |  |  |
| Hypertension | 445(66.02) | 340(66.27) | 0.008 | 0.927 |
| Diabetes mellitus | 135(20.03) | 109(21.25) | 0.265 | 0.607 |
| Atrial fibrillation | 101(14.99) | 88(17.15) | 1.023 | 0.312 |
| Coronary artery disease | 142(21.07) | 114(22.22) | 0.229 | 0.632 |
| Current smoking | 270(40.06) | 213(41.52) | 0.258 | 0.612 |
| Current drinking | 142(21.07) | 117(22.81) | 0.516 | 0.472 |
| **Medication use history, n (%)** |  |  |  |  |
| Previous antiplatelet | 88(13.06) | 77(15.01) | 0.929 | 0.335 |
| Previous anticoagulation | 48(7.12) | 36(7.02) | 0.005 | 0.945 |
| Previous statin | 51(7.57) | 40(7.80) | 0.022 | 0.882 |
| Previous antihypertension | 264(39.17) | 210(40.94) | 0.379 | 0.538 |
| Previous hypoglycemic agents | 79(11.72) | 62(12.09) | 0.037 | 0.847 |
| **Stroke subtype, n (%)** |  |  | 1.574 | 0.814 |
| LAA | 222(32.94) | 174(33.92) |  |  |
| SAO | 313(46.44) | 231(45.03) |  |  |
| CE | 102(15.13) | 88(17.15) |  |  |
| SOE | 8(1.19) | 6(1.17) |  |  |
| SUE | 27(4.01) | 16(3.12) |  |  |
| **Laboratory data** |  |  |  |  |
| FBG (mmol/L) | 5.91(4.9-7.63) | 5.81(4.87-7.44) | -2.199 | 0.090 |
| TG (mmol/L) | 1.38(0.97-1.99) | 1.35(0.92-1.93) | -1.691 | 0.067 |
| TC (mmol/L) | 4.36(3.70-5.09) | 4.34(3.71-5.07) | -0.979 | 0.327 |
| HDL-C (mmol/L) | 1.10±0.26 | 1.12±0.40 | -1.135 | 0.257 |
| LDL-C (mmol/L) | 2.72±0.84 | 2.77±0.99 | -0.805 | 0.421 |
| TyG index | 7.21(6.84-7.71) | 7.14(6.69-7.58) | -1.824 | 0.074 |
| TG/HDL-C | 1.33(0.85-2.09) | 1.23(0.79-1.97) | -1.737 | 0.082 |

Abbreviations: A, Changsha central hospital; B, Hunan Province's Second People's Hospital; BMI, body mass index; sICH, symptomatic intracranial hemorrhage; SBP, systolic blood pressure; DBP, diastolic blood pressure; NIHSS, National Institutes of Health Stroke Scale; OTT, onset to treatment time; LAA, large-artery atherosclerosis; SAO, small-artery occlusion; CE, cardioembolism; SOE, stroke of other determined etiology; SUE, stroke of undetermined etiology; TC, total cholesterol; TG, triglycerides; FBG, fasting blood glucose; HDL-C, high-density lipoprotein cholesterol; LDL-C, low-density lipoprotein cholesterol; TyG, triglyceride-glucose.
